# Population pharmacokinetic modelling of rupatadine solution in 6–11 year olds and optimisation of the experimental design in younger children

**DOI:** 10.1371/journal.pone.0176091

**Published:** 2017-04-18

**Authors:** Eva Santamaría, Javier Alejandro Estévez, Jordi Riba, Iñaki Izquierdo, Marta Valle

**Affiliations:** 1 Clinical Development, R&D, J. Uriach y Compañía, S.A., Barcelona, Spain; 2 Departament de Farmacologia, de Terapèutica i de Toxicologia, Universitat Autònoma de Barcelona, Barcelona, Spain; 3 Pharmacokinetic/Pharmacodynamic Modeling and Simulation, CIM-St Pau, Institut de Recerca de l’Hospital de la Santa Creu i Sant Pau-IIB Sant Pau, Barcelona, Spain; 4 Human Neuropsychopharmacology Group, CIM-St Pau, Institut de Recerca de l’Hospital de la Santa Creu i Sant Pau-IIB Sant Pau, Barcelona, Spain; University of Kentucky, UNITED STATES

## Abstract

**Aims:**

To optimise a pharmacokinetic (PK) study design of rupatadine for 2–5 year olds by using a population PK model developed with data from a study in 6–11 year olds. The design optimisation was driven by the need to avoid children’s discomfort in the study.

**Methods:**

PK data from 6–11 year olds with allergic rhinitis available from a previous study were used to construct a population PK model which we used in simulations to assess the dose to administer in a study in 2–5 year olds. In addition, an optimal design approach was used to determine the most appropriate number of sampling groups, sampling days, total samples and sampling times.

**Results:**

A two-compartmental model with first-order absorption and elimination, with clearance dependent on weight adequately described the PK of rupatadine for 6–11 year olds. The dose selected for a trial in 2–5 year olds was 2.5 mg, as it provided a Cmax below the 3 ng/ml threshold. The optimal study design consisted of four groups of children (10 children each), a maximum sampling window of 2 hours in two clinic visits for drawing three samples on day 14 and one on day 28 coinciding with the final examination of the study.

**Conclusions:**

A PK study design was optimised in order to prioritise avoidance of discomfort for enrolled 2–5 year olds by taking only four blood samples from each child and minimising the length of hospital stays.

## Introduction

The pharmacokinetics (PK) and pharmacodynamics of many medications, including those used in the treatment of allergic diseases, have not been optimally investigated in paediatric populations [1]. Clinical trials in children present several challenges: only a small pool of child patients is available, primary care doctors and/or parents are reluctant to enrol young patients, and children have certain attitudes and requirements related to comfort. Children dislike blood extraction, for example, bad-tasting medicines and interruptions in their routines [2]. However, these difficulties should not stand in the way of research into appropriate medicines and/or formulations in this population, as children have the right to benefit from safe and efficacious treatments. The International Conference on Harmonisation (ICH) for Requirements for Registration of Pharmaceuticals for Human Use has therefore issued guidelines [3] on the design of paediatric clinical trials to collect PK/pharmacodynamic, efficacy and safety data.

Pain and anxiety related to the invasiveness of extraction and the volume of blood drawn as well as ethical considerations in the paediatric population are responsible for limited blood samples, and preclude the use of classical approaches to studies in children. Population PK approach is a good alternative since it can deal with sparse data. [4,5]. However, it is crucial to design the experiment in such a way as to avoid the occurrence of imprecise and unreliable results, which lead to erroneous conclusions. Methods based on optimal design theory can lead to true optimisation of trial design [6] and have been used in the past for this purpose in other scenarios [7–9].

Rupatadine, a second generation antihistamine with anti-H1 and anti-platelet-activating factor antagonist activity [10], has proven effective in relieving allergic rhinitis and urticaria symptoms [10]. No cardiac safety effects (QTc interval prolongation) were detected in a 5-day repeated dose study done in healthy adults according to ICH recommendations (ICH E14) [11] at doses of 10 and 100 mg (10-fold the authorised dose in adults) [12]. When the safety profile was evaluated in several central nervous system or interaction studies and in a long-term (1-year) safety study, no concerns were raised by reports of adverse events [13].

The daily authorised dose in adults and adolescents (>12 years old) for the treatment of allergic rhinitis and urticaria is 10 mg in tablet form. The authorised dose in children for the same indication is 5 mg if weight equal or higher than 25 kg and 2.5 mg if weight is lower than 25 kg. In adults, rupatadine is rapidly absorbed, reaching a mean maximum concentration of 2.6 ng/ml 45 min after oral intake of a 10-mg tablet. Protein binding is around 98.5–99%, and the elimination half-life is 5.9 hours [14]. Rupatadine is mainly metabolised by CYP3A4, the reason why its administration with strong CYP3A4 inhibitors should be avoided. However, it can be taken with or without food. Exposure in elderly volunteers has been observed to be increased but no clinical significance has been noted. Limited PK data are available for rupatadine in children, however. The objective of the present study was to optimise a PK study design for 2–5 year olds by using a PK model we developed with data from a population of 6–11 year olds. The design optimisation was driven by the need to avoid the children’s discomfort during the study.

## Material and methods

### Population

PK data from an open label, single dose study in eleven children with allergic rhinitis aged 6–11 years old were used to build the model. The study was approved by the Clinical Research Ethics Committee of the Royal Children’s Hospital and Murdoch Children’s Research Institute, Melbourne, Australia, and by the Ethics Committee of the Peninsula Private Hospital and Peninsula Clinical Research Centre, Rivercity, Australia. The study protocol was also authorised by the Department of Health and Ageing, Therapeutic Goods Administration, of the Australian Government (study number 2006/593). Parents or guardians of the enrolled children gave their consent to participation in the study and signed an informed consent form before any study procedure took place.

Children 6–11 years of age, weighing ≥16 kg, were eligible for enrolment. They had a history of allergic rhinitis and were in good health. Participants who had taken any medications that could significantly interact with CYP3A4 were excluded, as rupatadine is mainly metabolised by this enzyme. A full concentration-time profile was obtained for each child after oral administration of a rupatadine 1 mg/ml solution. A total of 8 blood samples were drawn at the following times: predose, and at 0.5, 1, 2, 4, 8, 12 and 24 hours postdose. We administered 2.5-ml oral doses of the rupatadine 1 mg/ml solution in children weighing >10 and <25 kg, and 5-ml doses in children weighing ≥25 kg.

### Analytical method

Blood samples (4 ml) were collected in lithium-heparin tubes at each time-point. Afterwards the samples were centrifuged at approximately 3000 rpm for 10 min at –4°C. Supernatant plasma was then separated with Pasteur pipettes in 2 aliquots of 1 ml each. Both aliquots were carefully transferred to a collection tube and frozen below –20°C until analysis.

Plasma levels of rupatadine were measured using a validated liquid chromatography–mass spectrometry (MS)/MS analytical method. Briefly, clomipramine (internal standard) was added to plasma samples followed by extraction with terc-butyl methyl ether. The analysis was performed with an API 4000 spectrometer using a Columbus C18 column (50 mm × 4.6 mm × 5 μm) with a gradient mobile phase of 0.2 M ammonium acetate pH 4.5/acetonitrile, pumped at 1 ml/min. The linear range of the assays was 0.1 to 10 mg/l and the lower limit of quantification was 0.1 mg/l. The within- and between-run precision error was less than 12.71%. Accuracy-related errors were within ±12.83% for this rupatadine-selective method.

### Population analysis

One- and two-compartment disposition models with first-order absorption were fitted to the data using the first-order conditional estimation (FOCE) procedure by means of NONMEM software [15] using a Fortran compiler (version 6).

The tested models were described in terms of volume of distribution of the central compartment (Vc/F); volume of distribution of the peripheral compartment (Vp/F); total body clearance (CL/F); intercompartmental clearance (CLd/F); and the absorption rate constant (ka). In addition, the inclusion of absorption lag time (Tlag) was evaluated in order to help adjust the model to the data in the absorption phase.

Interindividual variability of the parameters was estimated assuming a proportional variance model, with exponential errors following a log-normal distribution, as shown in the following equation:

CLi=CLpop·exp(ηi)

where *CLi* is the true PK parameter of the *i*^th^ individual, *CLpop* the population value for the typical individual, *ηi* is the interpatient random effect with a mean of 0 and variance *ω*^2^.

Additive, proportional and combined residual error models were tested in order to find which best described residual variability.

Possible correlations between covariates were evaluated graphically. If two covariates were correlated, the one with more promising clinical applications was selected to be further tested in NONMEM. The relationships between covariates and Bayesian estimated parameters from the selected basic model were explored graphically. A forward inclusion and backward elimination approach was planned for determining which covariates to include in the model. The investigated covariates were: age, sex, weight, height and body mass index (BMI).

For considering a model superior to other nested models, the following aspects were taken into account: the reduction of the objective function (OF) value (provided by NONMEM) by at least 3.84 points (P < 0.05, one degree of freedom; approximate χ^2^ distribution), a lower estimation error in the parameters, visual judgement of goodness of fit using S-PLUS software for graphical displays, the convergence of the model, and the covariance matrix. During the covariate inclusion we used a P < 0.1 due to the small sample size, if there was an improvement in the other points mentioned above.

As no additional data from another study was available to use an external validation of the model, when the model was developed, we evaluated its performance by means of a visual predictive check using Monte Carlo simulations, which is a well accepted approach [16]. One thousand rupatadine concentration-time profiles were simulated after the administration of a single dose. These profiles were generated using the fixed and random estimates obtained from the final selected model. The mean profile and the intervals including 90% of the simulated concentrations (empiric bayesian estimates, EBEs) were plotted together with the raw data. The agreement between simulations and observations was judged visually.

### Dose and study design simulations

Different scenarios were simulated in order to decide the best dose, of the same rupatadine oral solution formulation used in 6–11 year old, to be administered to 2–5 year olds, when designing a PK study in that age group.

#### Dose selection

Children 2–5 years of age weight ranges from 10 to 25 kg according to growth charts of the Centers for Disease Control and Prevention [17]. Thus, using our final PK model for 6–11 year olds, we performed simulations for children weighting 10, 15, 20, and 24 kg. The two doses used for the simulations were 2.5 and 5 mg. Children weighing equal or greater than 25 kg were not simulated as that weight is rare and the study in 6–11 year olds clearly indicated that a dose of 5 mg should be administered in the heavier children.

#### Optimal design of the next study in 2–5 year olds

The final model was also used to explore the optimal design for a PK study in 2–5 year olds. It was assumed that PK in this age group would be similar to the PK observed in 6–11 year olds. Based on the simulations a dose of 2.5 mg was selected if weight was lower than 25 kg, and 5 mg if equal or greater to 25 kg.

The optimal number of sampling time-points was assessed using WinPOPT software (Release 1.2.1) [18]. The selection of the best design was based on statistical and child factors including the length of time the children were admitted to the hospital’s research facilities on the days required to carry out the study. WinPOPT provides the following statistics: the determinant, the relative optimality criterion between two models (efficiency), and the standard errors for the fixed parameters. The optimal design is the one that provides the highest value of the determinant. The optimality criterion, which is derived from the determinant, is the determinant to the power of 1 over the number of parameters in the model (1/P). The higher the optimality criterion, the more information is contained in the designed population study; therefore lower estimation errors would be expected. However, in this particular study, the well-being of the enrolled children played an important role in model selection; specifically, we sought a model that would require as short a hospital stay as possible.

Assuming a total sample of 40 children, different scenarios (Table 1) were studied, including different numbers of groups (2, 3 or 4 groups), sampling on days 14 or 28 or both, number of total samples per child (3 or 4), and different sampling times. Sampling time on day 28 was intended to be at the same time as the final examination.

**Table 1:** Schedule of simulated scenarios.

Number of groups	Days of sampling	Number of samples per day
2	28	4
	14,28	3,1
	14, 28	2,1
3	14	3 or 4
	28	4
	14,28	3, 1
	14,28	2,1
4	14,28	3, 1
	14, 28	2, 1

## Results

### Dataset

A dataset with concentration and time data from the eleven 6–11 year olds along with sex, age, weight, height and BMI was used to develop the model. The age distribution was as follows: 7 years (9.1%), 8 years (9.1%), 9 years (27.3%), 10 years (9.1%), and 11 years (45.4%). A total of 84 plasma concentrations of rupatadine were used in the analysis. Concentrations below the limit of quantification in the elimination phase (amounting to only 12% of observations) were not considered in the calculations [19]. The observed concentration range of rupatadine was 0.1 to 4.9 ng/mL. Most children received a 5-mg dose since all but 2 children weighed more than 25 kg. These two children received a 2.5 mg dose. Table 2 summarises the demographic characteristics of the included patients.

**Table 2:** Demographic characteristics of the 6–11 year olds included in the data analysis.

Variables (units)	Mean (SD)	Median (min–max)
*Gender (male/female)	5/6	–
Age (years)	10.23 (1.29)	10.41 (7.94–11.93)
Body weight (kg)	38.55 (14.31)	38.5 (22.0–68.5)
Height (m)	1.407 (0.128)	1.44 (1.18–1.59)
BMI (kg/m^2^)	18.83 (3.95)	18.54 (13.4–27.1)

*Total number instead of mean value is presented

SD, standard deviation; min, minimum; max, maximum; BMI, body mass index.

Rupatadine was well tolerated in the study and reported adverse events (mild headache and nausea) were similar to those reported in adults.

### Population analysis

**Fig 1:**
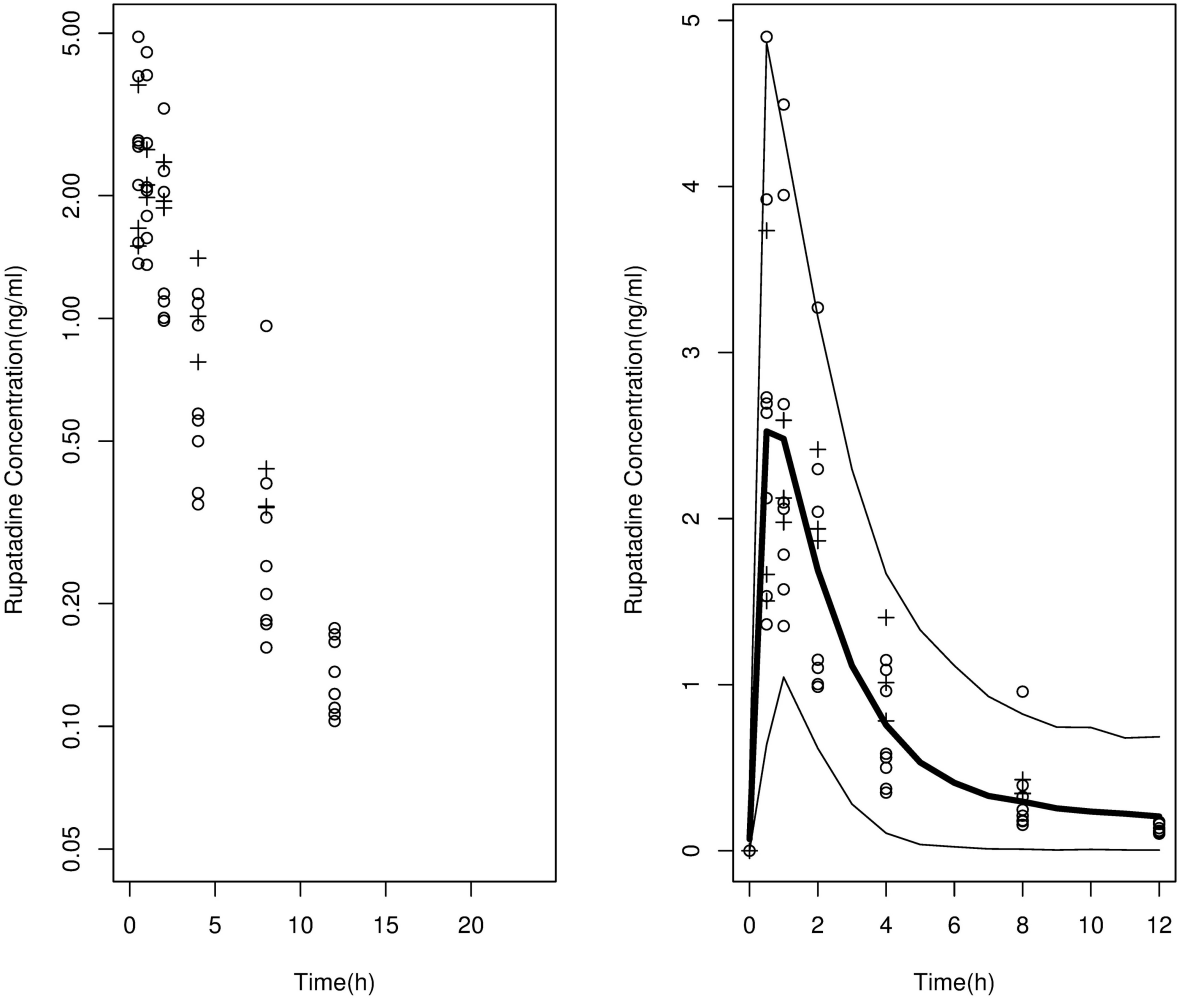
Time profile of the observed plasma concentrations of rupatadine in 6–11 year olds after the administration of 2.5-mg (crosses) or 5-mg (open circles) doses. Left panel, observed concentrations of rupatadine in log scale. Right panel, visual predictive check of the final selected model built with data for rupatadine in 6–11 year olds. Solid thin lines cover the area including 90% percentile interval of the simulated plasma concentrations over time, and thick line represents the mean of the simulated profiles. Concentrations have been normalised to a 5-mg dose.

Visual inspection of the concentration-time data (Fig 1) suggested that a one-compartment model with first-order absorption combined with an additive error model would provide the simplest description of the data; however on further evaluation it was seen to be biased toward concentrations measured at the end of the dosing interval. We therefore tested whether a two-compartment model would improve the fit. The two-compartment model with interindividual variability in CL And V provided a significant decrease of 13 points in the OF compared to the model with a single compartment of disposition (P< 0.005) and predictions that were closer to the observations. The incorporation of a lag time in the absorption process further improved the fit. The data only supported interpatient variability in CL/F and Vc/F. Residual variability was explained by means of an additive error model. Base model population parameter estimates are shown in Table 3. The inclusion of covariates was further studied in the base model.

**Table 3:** Estimates of the parameters from the base model and final selected model.

Parameters (units)	Base Model	Final Model
	Estimate (RSE) (RSE)	Estimate (RSE)
ka (h^–1^)	0.53 (15)	0.53 (15)
Lag time (h)	0.22 (18)	0.22 (13)
CL/F(L/h)	547 (15)	θ_1_ = 225 (63)
		θ_2_ = 333 (44)
Vc/F (L)	102 (59)	108 (52)
CLd/F (L/h)	208 (13)	209 (30)
Vp/F (L)	1540 (68)	1430 (56)
IIV CL/F (%)	45 (29)	40 (25)
IIV Vc/F (%)	94.6 (42)	93.8 (38)
Residual error (ng/mL)	0.19 (35)	0.18 (41)

ka, first order absorption rate constant; CL/F, apparent plasma clearance; Vc/F, apparent volume of distribution of the central compartment; CLd/F, apparent distribution clearance; Vp/F, apparent volume of distribution of the peripheral compartment. IIV, interpatient variability expressed as coefficient of variation; RSE, residual standard error.

CL/F for the final model: CL = θ1+ θ2*WEIGHT/38.5

### Covariate inclusion

As expected, there was a clear correlation between weight and height, so we decided to test weight and BMI for significance in addition to age. According to the plots, weight, BMI, and age all appeared to correlate with rupatadine clearance (data not shown). Therefore sex and height were not further evaluated in NONMEM. BMI was tested for significance in NONMEM, but no clear improvement of the model was detected respect to the base model (OFV = -114 vs -112, P>0.05). Although an allometric scaling model was tested, the function that best described the weight–CL/F relationship was a linear model (equation below).

$${\text{CLi}=\;\theta }_{\text{1}}{\;+\;\theta }_{\text{2}}*(\text{Weight}/38.5)$$

The incorporation of weight into the rupatadine clearance model decreased the value of the OF in 3 points (P = 0.08), generally improved the estimation errors with respect to the base model and explained 11.1% of the interindividual variability in CL/F. When the influence of weight on V/F was evaluated, the results argued against its inclusion in the model. Age was also tried in the CL/F in the base model, but the estimation error increased considerably. When age was included in the V/F model as univariate, there was no change in the OF and it was deemed appropriate to exclude age from the final model following the criterion of simplicity. The final model provided the PK parameters showed in Table 3.

### Model evaluation

The prediction capability of the final model was evaluated by means of Monte Carlo simulations. The 90% percentile interval of the 1000 simulated profiles included the majority of the empirical observations from the study of 6–11 year olds (Fig 1). Thus, the model was considered useful for predicting plasma concentrations.

### Simulations to select the dose for 2–5 year olds

**Fig 2:**
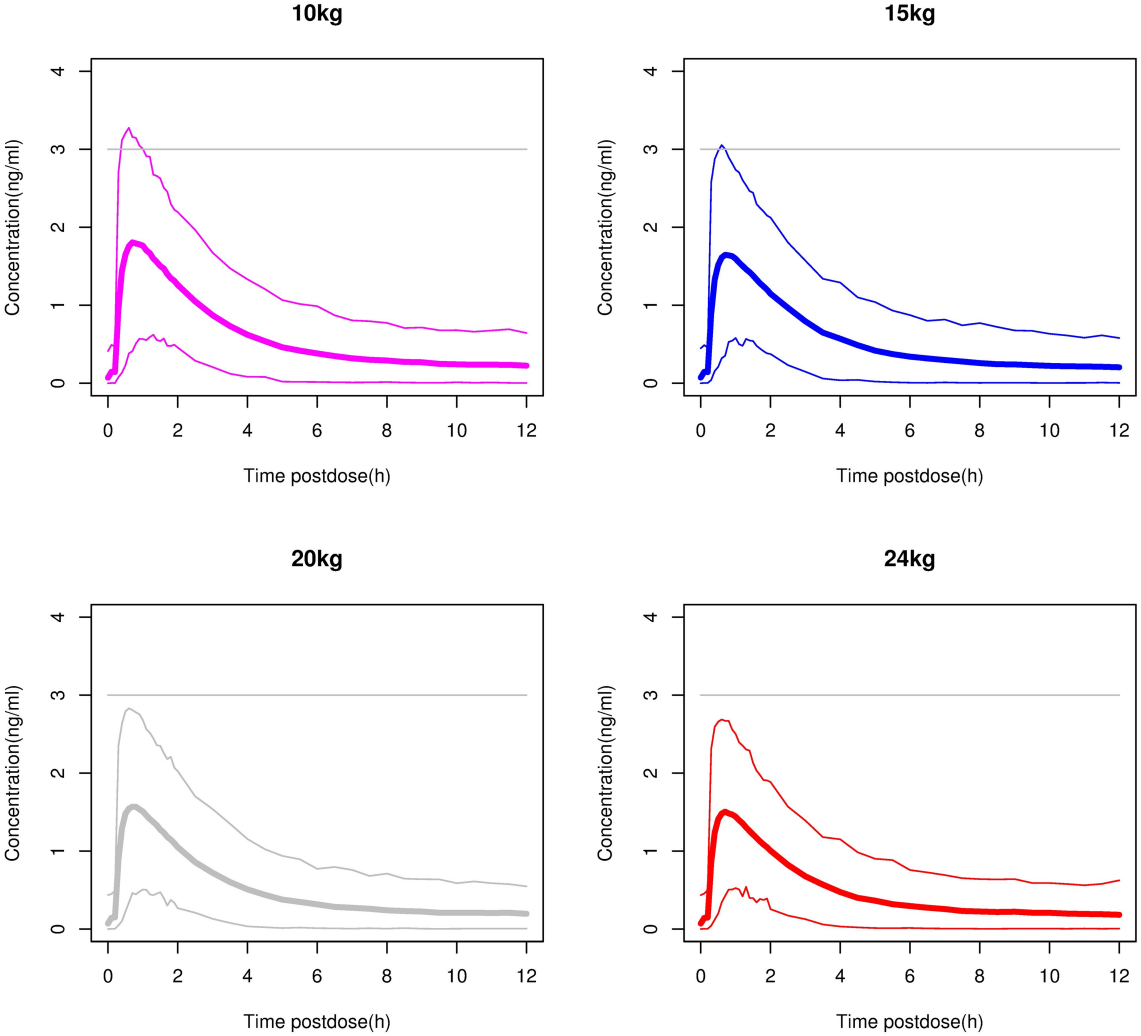
Simulations in children weighing 10, 15, 20 and 24 kg of after a single 2.5-mg dose of rupatadine 1 mg/ml oral solution. The thin lines mark the boundaries of the area including 98% of the simulated plasma concentrations over time (98% prediction interval), and the thick line represents the mean of the simulated profiles. The horizontal grey line represents the maximum concentration targeted in these children.

**Fig 3:**
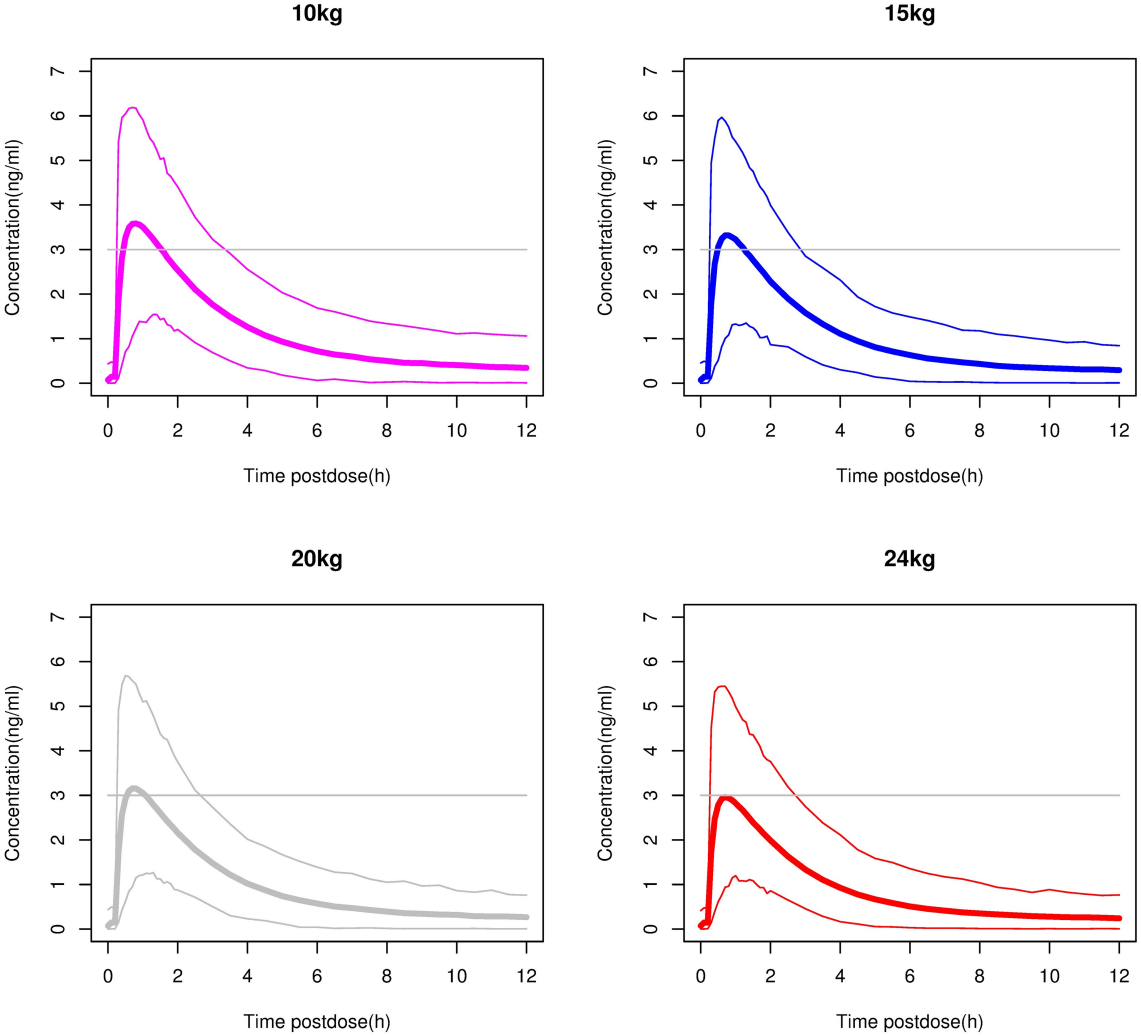
Simulations in children weighing 10, 15, 20 and 24 kg after a single 5-mg dose of rupatadine 1 mg/ml oral solution. The thin lines mark the boundaries of the area including 98% of the simulated plasma concentrations over time, and the thick line represents the mean of the simulated profiles. The horizontal grey line represents the maximum concentration targeted in these children.

Fig 2 shows the predicted profiles (mean and 98% prediction interval) for 2–5 year olds with different weights after they received a single oral dose of 2.5 mg. The Cmax for the majority of the children was below the 3 ng/ml threshold. In Fig 3 the simulated profiles of children receiving single 5-mg oral doses are shown. In this case a Cmax of >3 ng/ml would be achieved in more than half the children. Considering that the objective was to keep the Cmax below 3 ng/ml in the majority of children, a 2.5-mg dose was selected for testing in children weighing more than 10 kg and less than 25 kg.

### Design optimisation

The optimisation of the design for a rupatadine trial in 2–5 year olds was performed in several steps. Models of several blood sampling strategies were tested as described in Table 4. In the first step, a two-group design with 20 in each group and 4 samples taken on day 28 (model A) was tested. This scenario involved a long clinic stay (minimum 8 hours), a situation to be avoided as far as possible. For this reason, we evaluated several other designs with four groups instead of two and with samples taken on days 14 and 28. The length of the clinic stay shortened in model B, but this model involved attending twice to the hospital at day 28, once for PK sampling and another one to perform the final visit of the study. Therefore, although the determinant and the optimal criteria were good, 0.383 and 0.88 respectively, other models were tested in order to avoid such repeated visits to the hospital at the end of the study. By simply changing the time of drawing blood on day 28 to somewhere around 24 hours after the last dosing (model C), the estimation of the parameters improved over those estimated by model A and the conditions for the enrolled children on day 28 improved over those of model B. If the design reflected in model C were applied, the children would have to stay in the clinic for no longer than 3 hours on day 14. Next, we checked whether the duration of the clinic stay on day 14 could be reduced without compromising the estimation of parameters. The final model selected (model D) showed that similar results could be achieved if children stayed at the clinic for only 2 hours on day 14. In this way, the daily dose of rupatadine can be administered by parents to the child at home during the study.

**Table 4:** Design optimisation for a study in 2–5 year olds.

Model (sampling strategy)	Groups	N	Sampling day	Sampling times after drug administration (h)	Determinant	Optimal Criteria
A	1	20	Day 28	1, 4, 6, 8	0.008	0.55
	2	20	Day 28	8, 10, 12, 24		
B	1	10	Day 14	0.5, 1, 3	0.383	0.88
			Day 28	2		
	2	10	Day 14	4, 5, 6		
			Day 28	3		
	3	10	Day 14	7, 8, 9		
			Day 28	4		
	4	10	Day 14	10, 11, 12		
			Day 28	5		
C	1	10	Day 14	0.25, 0.5, 2.25	0.026	0.63
			Day 28	24.3		
	2	10	Day 14	3, 5.5, 6		
			Day 28	25		
	3	10	Day 14	6, 6.25, 8.5		
			Day 28	25		
	4	10	Day 14	9, 10, 11		
			Day 28	24.8-		
D*	1	10	Day 14	0.25, 0.5, 2.25	0.013	0.58
			Day 28	24.3		
	2	10	Day 14	4, 5.5, 6		
			Day 28	25		
	3	10	Day 14	6, 6.25, 7		
			Day 28	24.5		
	4	10	Day 14	8, 9, 10		
			Day 28	23		

* Selected model. The comparison of the optimal criteria from two different model provides the efficiency of one model with respect to the other.

## Discussion

The present study used a population PK approach to describe rupatadine PK in children 6 to 11 years old with allergic rhinitis. The model was then used to establish the most suitable dose of rupatadine for 2–5 year olds and the optimal PK study design in these younger children. The design chosen avoids drawing more than four blood samples per patient and while obtaining enough data for a rupatadine PK study in this age group.

The PK of rupatadine in 6–11 year olds after a single oral dose followed a bicompartmental model with first order absorption and elimination processes, as has been previously described for adults [20]. The parameters estimated for children are very similar to those reported for adults taking the authorised 10-mg tablet formulation, suggesting that no differences in the PK of rupatadine are likely between adults and children, other than the volume of distribution. The estimated volume of distribution for a typical child was half that estimated value for a typical adult, however, when corrected by body weight no differences were found; the same results have been obtained for other antihistamine drugs such as desloratadine or levocetirizine [21, 22]. Even though adults received tablets in the cited study and the children in our study received a rupatadine solution, there were no apparent differences in the absorption rate constant (0.432 h^-1^ estimated for adults by Peña *et al* [20] and 0.53 h^-1^ for children in our study). In neither study was it possible to estimate interindividual variability for the absorption process, probably due to the small number of patients included and/or the low number of samples during the ascending phase of the concentration-time curve. The difficulty in estimating interindividual variability in ka may account, at least in part, for the high interindividual variability in Vc/F. On the other hand, according to our final model, the estimated value of CL/F for a typical 6–11 year old weighting 38.5 kg would be 558 l/h, similar to the values of 467 l/h for men and 732l/h for females obtained for adults[20].

Clearance proved dependent on weight but not on age or sex. The lack of correlation between age and clearance was unsurprising considering the narrow range of age of the participants and the fact that CYP3A4, the main enzyme responsible for the metabolisation of rupatadine, reaches enzyme maturation at 1.3 years of age according to a recent re-evaluation of CYP3A4 ontogeny function [23]. Therefore, only size is expected to affect the clearance of many drugs, including rupatadine, after that age. Moreover, this result confirmed our dosing expectations that rupatadine PK would depend on a child’s weight based on the behaviour of other antihistamines. Fexofenadine given to children (30 mg if >10.5 kg and 15 mg if ≤ 10.5 kg) provided similar exposure levels to those found in adults in one study [24]. Similarly, weight influenced both CL/F and V/F in a population study of epinastine dry syrup in children aged 2–15 years old [25]. The authors recommended dosing according to body weight in children, but no effect of age was included in any of the model parameters for epinastine. Regarding cetirizine, age was the only covariate that explained part of the interindividual variability in CL/F and V/F in children [4], probably because that study enrolled very young participants (from the age of 6 months). Finally, although rupatadine clearance depends on gender in adults [20], we did not find this relationship in children, most likely because of the small size of our sample and because weight may be capturing differences between males and females.

Antihistamines are often prescribed for conditions that affect young children, especially allergic disorders—allergic rinithis and urticaria in particular. In order to optimise the selection of the dose to be administered in a trial in young children (2–5 year olds in our study), it is useful to predict the expected exposure and compare it with the exposure in adults [8]. The model developed in the present study was further used to predict this age group’s exposure in individuals with a normal weight range of 10–25 kg [17]. The model predicted a Cmax above 3 ng/ml in more than 50% of patients for a single daily dose of 5 mg. Although a study in adults has shown that concentrations above 3 ng/mL provide a good response to the drug without serious safety concerns in adults [10], we selected a conservative dose of 2.5 mg every 24 h, to provide the majority of patients with a Cmax in the 1–3 ng/ml range, which we foresee would be efficacious and safe in this age group.

We also used modelling to determine the optimal design for obtaining adequate information for studying rupatadine PK in 2–5 year olds while maximizing their well-being and comfort by means of carefully selected sampling times. The predicted differences in the PK parameters in the four scenarios presented in this paper were not great, allowing us to choose the ideal scenario based on the personal well-being of the children. The main benefit of the chosen model is that patients will not have to stay in the hospital’s research facility longer than 2 hours. This study demonstrates a clear advantage of using population PK modelling, simulation and optimal design approaches during the drug development process. The design obtained is in agreement with the Ethical Considerations for Clinical Trials performed in children [26], which counsels limiting not only the volume of blood drawn but also the pain and distress caused in children in clinical interventional studies. The less time children remain at the hospital, the less distress they feel.

The main limitation of the proposed methodology to optimise the sampling times for 2–5 year olds is that the Fisher information matrix relies on estimated parameters. Therefore, any misspecification in the model structure, and/or the estimation of the parameters during the model development phase would influence the results of design optimisation. A sensitivity analysis to detect model misspecification and or misspecification of parameter values was not performed during our development of the model with data from 6–11 year olds. This procedure is very time consuming and there is no clearly accepted way to do it in multivariate analyses [9]. Although we are confident about our model’s structure since full PK profiles were available for all the children, the interindividual variability estimated from the model means we should be cautious in our interpretation given the small number of participants and the fact that weight was not incorporated as covariate during the simulation process.

In conclusion, the PK of rupatadine in 6–11 year olds can be explained by a two-compartmental model with first-order absorption and elimination in which weight influences central compartment clearance. This model proved useful for optimising the dose and sampling times for a future study to evaluate the PK of rupatadine in 2–5 year olds. The selected design requires taking only a few samples (four from each child) and avoids a long hospital stay (a maximum of two hours), thus minimising children’s discomfort and distress.
